# Clinical Outcome of Paclitaxel-Coated Balloon Angioplasty Versus Drug-Eluting Stent Implantation for the Treatment of Coronary Drug-Eluting Stent In-Stent Chronic Total Occlusion

**DOI:** 10.1007/s10557-022-07363-7

**Published:** 2022-08-05

**Authors:** Yuchao Zhang, Zheng Wu, Shaoping Wang, Tong Liu, Jinghua Liu

**Affiliations:** Center for Coronary Artery Disease, Beijing Anzhen Hospital, Capital Medical University, Beijing Institute of Heart Lung and Blood Vessel Diseases, No. 2 Anzhen Road, Chaoyang District, Beijing, 100029 China

**Keywords:** Percutaneous coronary intervention, In-stent restenosis, Chronic total occlusion, Paclitaxel-coated balloon, Drug-eluting stent

## Abstract

**Aims:**

In-stent chronic total occlusion (IS-CTO) represents a unique challenge for percutaneous coronary intervention. Whether the optimal treatment for IS-CTO is angioplasty with paclitaxel-coated balloons (PCBs) or repeat stenting with drug-eluting stents (DESs) is unclear. We aimed to evaluate the long-term clinical outcome of PCB angioplasty and DES repeat stenting for DES IS-CTO.

**Methods:**

We retrospectively included patients with DES IS-CTO who underwent successful PCB angioplasty or DES repeat stenting from January 2016 to December 2019. The primary endpoints were major adverse cardiac events (MACEs), including cardiac death, myocardial infarction, and target lesion revascularization (TLR). Cox proportional hazards model was performed to compare the risk of MACEs between PCB angioplasty and DES repeat stenting, and to further explore the prognostic factors of patients with DES IS-CTO.

**Results:**

A total of 214 patients with DES IS-CTO were enrolled: 78 patients (36.4%) treated with PCB and 136 patients (63.6%) treated with DES respectively. The median follow-up was 1160 days, and MACEs were observed in 28.2% of patients with PCB angioplasty versus 26.5% of patients with DES repeat stenting (*P* = 0.784), mainly driven by TLR (21.8% vs. 19.9%, *P* = 0.735). There was no significant difference in the risk of MACEs between the PCB group and the DES group (hazard ratio [HR] 1.25, 95% confidence interval [CI] 0.64–2.46, *P* = 0.512). Multivariate Cox analysis revealed that chronic kidney disease and ≥ 3 stent layers in the lesion were independent predictors of MACEs, while switching to another antiproliferative drug was an independent protective factor (all *P* < 0.05).

**Conclusions:**

PCB angioplasty was an effective alternative treatment strategy for DES IS-CTO, which had similar long-term outcomes to DES repeat stenting in contemporary practice, but both were accompanied by a high rate of long-term MACEs. Improving the poor prognosis of patients with DES IS-CTO remains a challenge.

**Supplementary Information:**

The online version contains supplementary material available at 10.1007/s10557-022-07363-7.

## Introduction

The application of drug-eluting stents (DESs) is predominant in routine coronary intervention. Antiproliferative drugs bound to the surface of the DES can inhibit active neointimal hyperplasia after stent implantation. The new generation of DESs are especially effective and can further reduce the risk of restenosis and need for target lesion revascularization (TLR) [1, 2]. However, within 5 years of stenting with DESs, approximately 7 to 10% of patients undergo re-revascularization of the target lesion due to in-stent restenosis (ISR) [3–5]. In the field of percutaneous coronary intervention (PCI), ISR and chronic total occlusion (CTO) are two major challenges. In-stent chronic total occlusion (IS-CTO) is a kind of coronary artery disease that occurs after stenting and has characteristics of both ISR and CTO. The number of IS-CTO cases accounts for approximately 10 to 25% of all PCI performed for CTO [6–8]. Although the incidence of IS-CTO is not extremely high, due to the popularity of percutaneous coronary stenting, especially in the contemporary era, patients who have received DES implantation constitute a very large base, and the public health burden caused by DES IS-CTO cannot be underestimated.

Currently, the two main treatments for ISR are angioplasty with paclitaxel-coated balloons (PCBs) and repeat stenting with DESs [9–12]. However, current data are insufficient for comparisons of effectiveness between the two strategies. In this study, we compared the long-term clinical outcomes of patients who underwent angioplasty with PCB or repeat stenting with DES in the treatment of DES IS-CTO and identified the clinical predictors of MACEs, hoping to provide medical evidence for optimizing the treatment of DES IS-CTO in clinical practice.

## Methods

### Study Population

From January 2016 to December 2019, we retrospectively enrolled patients who underwent successful PCI with PCB or DES for DES IS-CTO in Beijing Anzhen Hospital. Patients treated with both PCBs and DESs at the lesion site and patients lost to follow-up were excluded in the final analysis. For patients with multiple interventions or multivessel disease, only the first successful PCI for DES IS-CTO was recorded. In each procedure, lesions were fully predilated with noncompliant balloons, scoring balloons, or cutting balloons. The choices of devices and implants were decided by the operators or interventional cardiologist teams. According to the different intervention treatments used at the occlusion site, the patients were divided into two groups: the PCB angioplasty group and the DES repeat stenting group. After patients were discharged from the hospital, professionally trained and experienced investigators obtained patients’ clinical endpoints through review of medical records or telephone interviews. Variable data were assessed independently by at least two cardiologists, and controversial data were submitted to a panel of independent experts (three certified cardiologists) for adjudication. Clinical, procedural, and outcome data were recorded by independent investigators in a dedicated database. The study protocol was performed according to the principles of the Declaration of Helsinki and approved by the hospital ethics committee.

### Definitions and Endpoints

CTO was defined as a coronary obstruction with a thrombolysis in myocardial infarction (TIMI) flow grade 0 for at least 3 months [13]. The occlusion duration was estimated by typical symptoms, angiogram, history of myocardial infarction in the target vessel territory, and new ischemic or infarct changes on electrocardiogram after the previous stent implantation. The CTO was considered in-stent if the occlusion was located within a previously deployed stent or within the 5-mm margins proximal or distal to the stent [14]. Procedural success was defined as successful CTO revascularization, achievement of < 30% residual diameter stenosis within the target lesions, and restoration of TIMI grade 3 flow, with no in-hospital serious adverse events, including death, myocardial infarction (MI), urgent target vessel revascularization with PCI or bypass surgery, cardiac tamponade requiring intervention or surgery, and stroke. The baseline demographic, angiographic, and procedure data were obtained through the Hospital Information System. The definition of high bleeding risk was based on the standards developed by the Academic Research Consortium for High Bleeding Risk [15]. The J-CTO score was calculated from 5 dimensions of blunt stump, calcification, bending > 45°, CTO lesion length ≥ 20 mm, and reattempt [16]. The PROGRESS CTO score was calculated from the 4 dimensions of proximal cap ambiguity, absence of interventional collaterals, moderate or severe tortuosity, and circumflex CTO [17]. The J-CTO score and PROGRESS CTO score were used to quantify the complexity of CTO lesions. The collateral circulation of CTO was graded with Rentrop classification [18]. The primary endpoints were major adverse cardiac events (MACEs), defined as a composite of cardiac death, MI, and TLR on follow-up [19]. Cardiac death was defined as any death of cardiac cause, unwitnessed death, or death without another known cause [20]. MI was defined according to the fourth universal definition of MI [21]. TLR was defined as any repeat percutaneous intervention or target vessel bypass surgery performed for restenosis or other complications of CTO target lesion [20].

### Statistical Analysis

Continuous variables are presented as the mean ± standard deviation (SD) or the median with first quartile (*Q*_1_) and third quartile (*Q*_3_). Student’s *t* test or the Wilcoxon rank-sum test were used to compare differences in continuous variables between groups. Categorical variables are presented as numerical values and percentages, and data were compared using the chi-square or Fisher’s exact test. To determine whether the application of PCB angioplasty or DES repeat stenting was associated with adverse long-term outcomes, survival curves were plotted to estimate the cumulative incidence of clinical events using the Kaplan–Meier method, and intergroup comparisons were performed using the log-rank test. The propensity score–based inverse probability of treatment weighting (IPTW) method was also performed to reduce possible selection bias and then evaluate the association between different intervention treatments and clinical outcome. Covariate include male, age, current smoker, diabetes, chronic kidney disease (CKD), high bleeding risk, calcification, CTO lesion length ≥ 20 mm, moderate or severe tortuosity, ostial lesion, proximal cap side-branch, diseased distal landing zone, time since last stents implantation in target lesion, and number of stent layers in lesions [11, 12, 15, 22–24]. After IPTW, weighted standardized mean differences of each covariate with values < 0.10 indicated a reasonable balance. The hazard ratios (HRs) of MACE were calculated using the Cox proportional hazards model. Univariable models were constructed for the treatments and other variables, including those with *P* values < 0.10 between participants with and without MACEs and between participants who underwent different treatments, other variables that incorporated into the propensity score model were also included. Further, a multivariable Cox model was constructed to adjust for variables with a *P* value < 0.10 in the univariable model. A *P* value < 0.05 on the two-sided test was considered statistically significant. All statistical analyses were performed using SPSS software, Version 22.0 (IBM Corporation, Armonk, NY, USA) and R statistical package version 4.0.3 (R Project for Statistical Computing, Vienna, Austria).

## Results

### Patient Characteristics

**Fig. 1:**
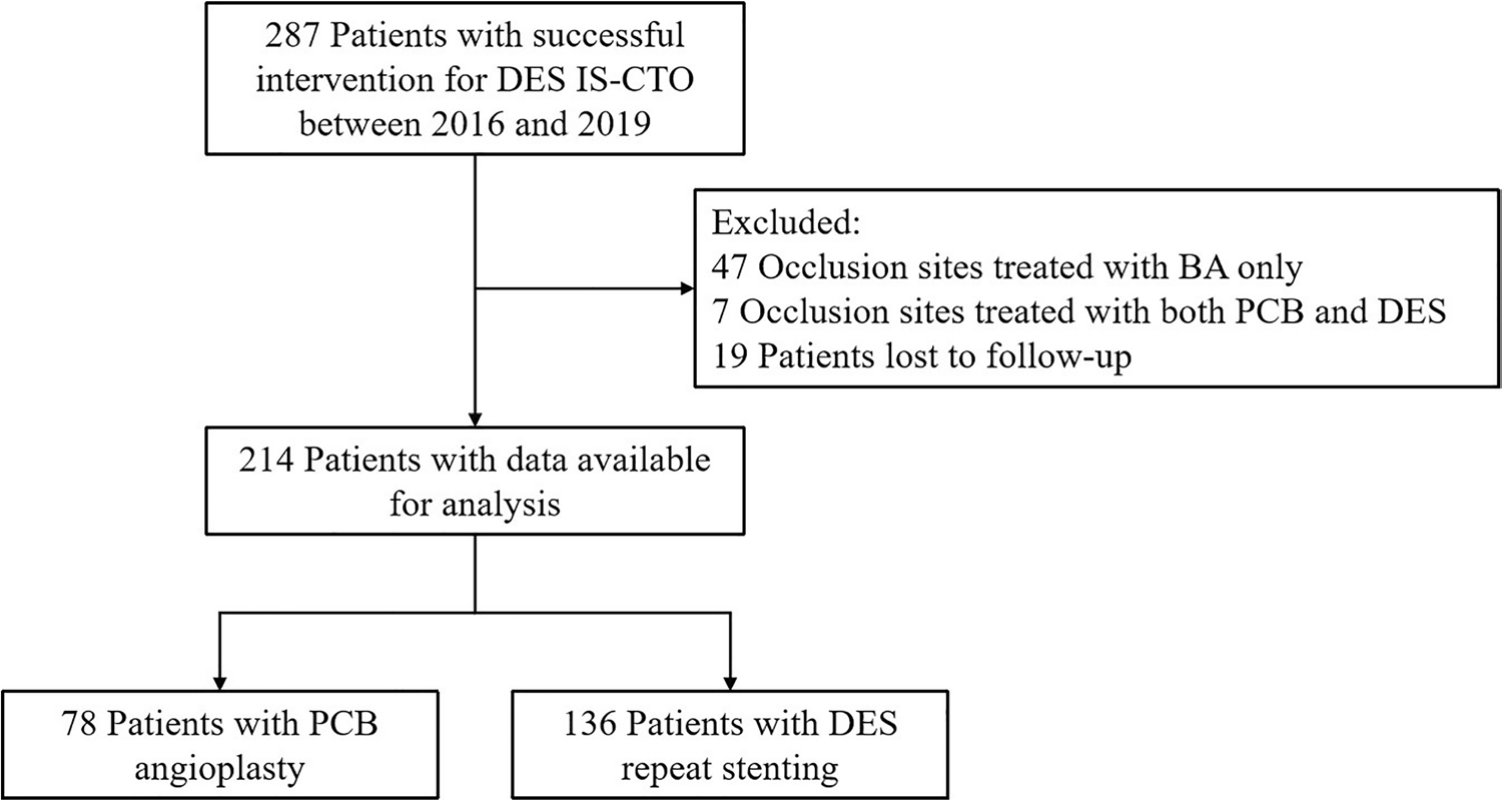
Flow chart of the study. BA, balloon angioplasty; DES, drug-eluting stent; IS-CTO, in-stent chronic total occlusion; PCB, paclitaxel-coated balloon

A total of 214 eligible patients were enrolled in our study, and the study flow chart is presented in Fig. 1. Seventy-eight patients (36.4%) were treated with PCB angioplasty, and 136 patients (63.6%) were treated with DES repeat stenting. The baseline demographic and clinical characteristics of the PCB angioplasty group and the DES repeat stenting group are presented in Table 1. A total of 83.6% of the patients were male, with a mean age of 59.1 ± 8.9 years. Baseline cardiovascular risk factors did not differ significantly between participants who underwent different treatments except for a higher ejection fraction in patients who underwent PCB angioplasty (62 (58–66)% vs. 59 (53–65)%, *P* = 0.016).

**Table 1:** Demographic and clinical characteristics

	PCB (*n* = 78)	DES (*n* = 136)	*P*-value
Male, *n* (%)	62 (79.5)	117 (86.0)	0.213
Age, years	57.8 ± 9.0	60.0 ± 8.8	0.097
Body mass index, kg/m^2^	26.5 ± 3.2	26.3 ± 2.8	0.558
Current smoker, *n* (%)	29 (37.2)	39 (28.7)	0.199
Hypertension, *n* (%)	50 (64.1)	84 (61.8)	0.734
Diabetes, *n* (%)	33 (42.3)	61 (44.9)	0.718
Dyslipidemia, *n* (%)	60 (76.9)	103 (75.7)	0.844
CKD, *n* (%)	10 (12.8)	9 (6.6)	0.125
Previous MI, *n* (%)	35 (44.9)	78 (57.4)	0.078
Previous CABG, *n* (%)	3 (3.8)	7 (5.1)	0.750
Ejection fraction, %	62 (58, 66)	59 (53, 65)	0.016
Total cholesterol, mmol/l	3.61 (3.17, 4.22)	3.82 (3.23, 4.30)	0.225
LDL cholesterol, mmol/l	2.01 (1.62, 2.57)	2.09 (1.79, 2.66)	0.258
HDL cholesterol, mmol/l	1.02 (0.88, 1.18)	1.01 (0.87, 1.17)	0.937
Triglycerides, mmol/l	1.25 (0.99, 1.79)	1.51 (1.06, 2.10)	0.088
Creatinine clearance, ml/min	102.38 ± 26.61	99.63 ± 26.58	0.468
High bleeding risk, *n* (%)	15 (19.2)	16 (11.8)	0.159
Medications, *n* (%)			
DAPT	77 (98.7)	135 (99.3)	> 0.999
Statin	78 (100.0)	135 (99.3)	> 0.999
β-Blocker	54 (69.2)	103 (75.7)	0.300
ACE-inhibitor/ARB	39 (50.0)	59 (43.4)	0.350

Data are presented as the mean ± standard deviation, *n* (%) or median (Q1, Q3)

*ACE* angiotensin-converting enzyme, *ARB* angiotensin receptor blocker, *CABG* coronary artery bypass graft, *CKD* chronic kidney disease, *DAPT* dual antiplatelet therapy, *DES* drug-eluting stent, *HDL* high-density lipoprotein, *LDL* low-density lipoprotein, *MI* myocardial infarction, *PCB* paclitaxel-coated balloon

### Angiographic Characteristics

The angiographic features of the two groups are shown in Table 2. The distribution of CTO target vessels was observed to be different between the two groups. PCB angioplasty was more frequently performed in the left anterior descending artery, while DES repeat stenting was more often performed in the right coronary artery IS-CTO. The J-CTO score (2 (1, 3) vs. 2 (1, 3), *P* = 0.855) and PROGRESS CTO score (1 (0, 1) vs. 1 (0, 2), *P* = 0.336) representing CTO complexity in the PCB group were comparable to those in the DES group. In our study, the time since last stent implantation in target lesion was 72 (interquartile range 37 to 109) months, and sirolimus-eluting stents were implanted in 150 (70.1%) patients during the previous PCI.

**Table 2:** Angiographic characteristics

	PCB (*n* = 78)	DES (*n* = 136)	*P*-value
Multivessel disease, *n* (%)	44 (56.4)	82 (60.3)	0.578
CTO target vessel, *n* (%)			0.001
Left anterior descending artery	44 (56.4)	43 (31.6)	
Left circumflex artery	5 (6.4)	21 (15.4)	
Right coronary artery	29 (37.2)	72 (52.9)	
J-CTO score	2 (1, 3)	2 (1, 3)	0.855
Blunt stump, *n* (%)	43 (55.1)	79 (58.1)	0.674
Calcification, *n* (%)	32 (41.0)	50 (36.8)	0.537
Bending > 45°, *n* (%)	22 (28.2)	47 (34.6)	0.339
CTO lesion length ≥ 20 mm, *n* (%)	59 (75.6)	95 (69.9)	0.364
Reattempt, *n* (%)	11 (14.1)	30 (22.1)	0.155
PROGRESS CTO score	1 (0, 1)	1 (0, 2)	0.336
Proximal cap ambiguity, *n* (%)	19 (24.4)	35 (25.7)	0.823
Absence of interventional collaterals, *n* (%)	30 (38.5)	48 (35.3)	0.643
Moderate or severe tortuosity, *n* (%)	10 (12.8)	27 (19.9)	0.190
Circumflex CTO, *n* (%)	5 (6.4)	21 (15.4)	0.052
Ostial lesion, *n* (%)	40 (51.3)	60 (44.1)	0.312
Proximal cap side-branch, *n* (%)	35 (44.9)	49 (36.0)	0.202
Diseased distal landing zone, *n* (%)	30 (38.5)	65 (47.8)	0.186
Collateral 2–3 grade, *n* (%)	56 (71.8)	93 (68.4)	0.601
Time since last stents implantation in target lesion, months	63 (36, 109)	78 (40, 110)	0.435
Prior type of DES, *n* (%)			0.230
Sirolimus-eluting stent	48 (61.5)	102 (75.0)	
Everolimus-eluting stent	10 (12.8)	11 (8.1)	
Zotarolimus-eluting stent	16 (20.5)	18 (13.2)	
Paclitaxel-eluting stent	4 (5.1)	5 (3.7)	
Prior stent length, mm	33 (24, 47)	32 (23, 47)	0.359
Prior minimum stent diameter, mm	2.75 (2.50, 3.00)	2.75 (2.50, 3.00)	0.662
Prior maximum stent diameter, mm	3.00 (2.75, 3.00)	3.00 (2.75, 3.50)	0.699
Prior mean stent diameter, mm	2.75 (2.75, 3.00)	2.88 (2.63, 3.00)	0.638
Number of stent layers in lesions, *n* (%)			0.019
1 layer	36 (46.2)	89 (65.4)	
2 layers	33 (42.3)	39 (28.7)	
≥ 3 layers	9 (11.5)	8 (5.9)	

Data are presented as *n* (%) or median (Q1, Q3)

*CTO* chronic total occlusion, *DES* drug-eluting stent, *PCB* paclitaxel-coated balloon

### Procedural Characteristics

In terms of procedure characteristics, although the final implants were different, other data between the two groups were basically balanced, as shown in Table 3. In our study, antegrade wire escalation was the main initial strategy. In the final successful strategy, although the proportion of antegrade wire escalation decreased, retrograde wire escalation or antegrade dissection re-entry accounted only for a small part. Furthermore, no significant differences were observed between the PCB group and the DES group in procedure time, fluoroscopy time, or contrast volume, which reflected the difficulty of the operation. When the effects of different antiproliferative drugs were considered, a significant difference was observed between the two groups in switching to another antiproliferative drug. The majority of the PCB group adopted a switch strategy, while the proportion was almost double that of the DES group (94.9% vs. 50.0%, *P* < 0.001).

**Table 3:** Procedural characteristics

	PCB (*n* = 78)	DES (*n* = 136)	*P*-value
Radial access, *n* (%)	64 (82.1)	120 (88.2)	0.210
Dual catheter injection, *n* (%)	22 (28.2)	53 (39.0)	0.112
Primary strategy, *n* (%)			0.750
AWE	75 (96.2)	129 (94.9)	
RWE	3 (3.8)	7 (5.1)	
Final strategy, *n* (%)			0.140
AWE	75 (96.2)	121 (89.0)	
ADR	0 (0)	4 (2.9)	
RWE	3 (3.8)	11 (8.1)	
Use of CrossBoss, *n* (%)	5 (6.4)	5 (3.7)	0.502
Use of IVUS, *n* (%)	10 (12.8)	15 (11.0)	0.695
Use of OCT, *n* (%)	5 (6.4)	3 (2.2)	0.144
Total procedure time, min	84 (67, 101)	89 (61, 112)	0.553
Total fluoroscopy time, min	28 (22, 39)	30 (21, 42)	0.594
Total contrast volume, ml	160 (140, 205)	170 (140, 208)	0.937
Number of PCB, *n*	1 (1, 2)		
Total PCB length, mm	30 (30, 60)		
Minimum PCB diameter, mm	2.75 (2.50, 3.00)		
Maximum PCB diameter, mm	3.00 (2.50, 3.50)		
Mean PCB diameter, mm	3.00 (2.50, 3.00)		
Maximal PCB pressure, atm	10 (8, 12)		
Duration of inflation, seconds	60 (49, 60)		
Number of DES, *n*		2 (1, 2)	
Type of DES, *n* (%)			
Sirolimus-eluting stent		84 (61.8)	
Everolimus-eluting stent		35 (25.7)	
Zotarolimus-eluting stent		17 (12.5)	
Total stent length, mm		55 (33, 73)	
Minimum stent diameter, mm		2.50 (2.50, 3.00)	
Maximum stent diameter, mm		3.00 (2.75, 3.50)	
Mean stent diameter, mm		2.75 (2.63, 3.13)	
Different antiproliferative drugs, *n* (%)	74 (94.9)	68 (50.0)	<0.001

Data are presented as *n* (%) or median (Q1, Q3)

*AWE* antegrade wire escalation, *ADR* antegrade dissection re-entry, *DES* drug-eluting stent, *IVUS* intravascular ultrasound, *OCT* optical coherence tomography, *PCB* paclitaxel-coated balloon, *RWE* retrograde wire escalation

**Fig. 2:**
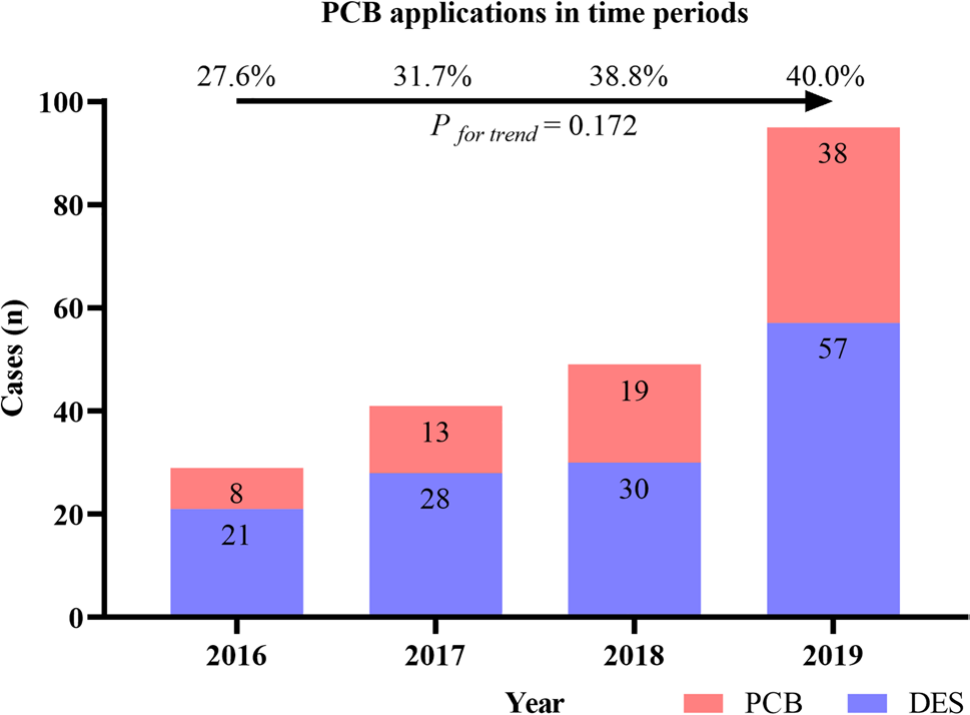
The number of successful cases and the corresponding proportion of PCB angioplasty in different time periods. DES, drug-eluting stent; PCB, paclitaxel-coated balloon

The number of patients who underwent successful IS-CTO PCI during the study period and the trend in the use of PCB angioplasty are presented in Fig. 2. Over time, the application of PCB angioplasty was observed to increase gradually, from 27.6% in the early period to 40.0% recently but no statistically significant difference (*P*_for trend_ = 0.172).

**Fig. 3:**
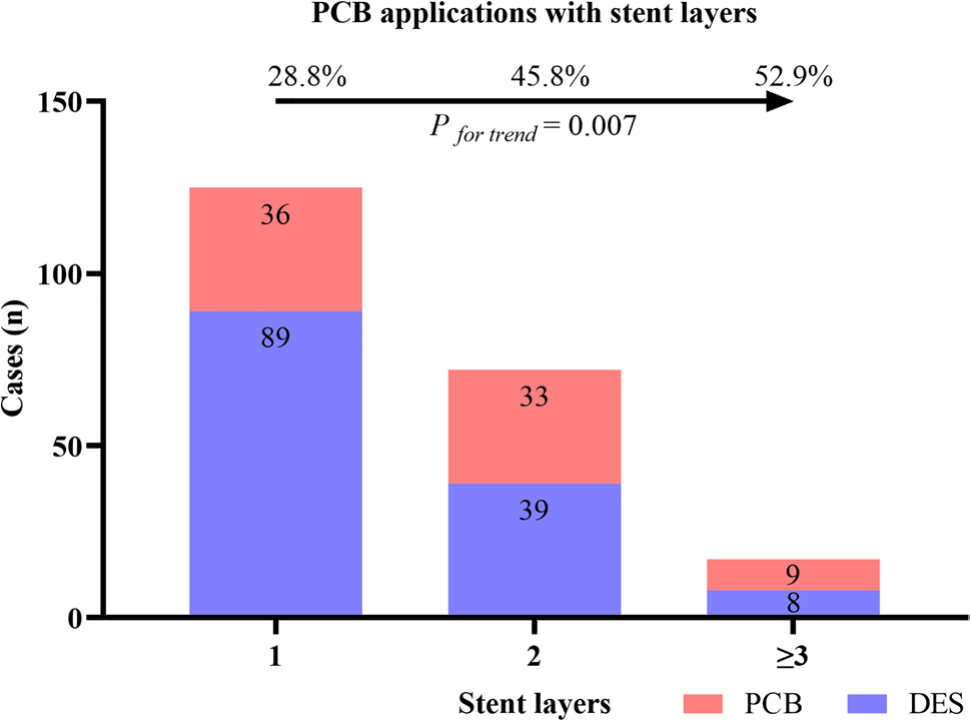
With the increase of stent layers, the application of PCB angioplasty. The proportion of different stent layers at the lesions and the corresponding treatments are shown in the figure. DES, drug-eluting stent; PCB, paclitaxel-coated balloon

Furthermore, when we grouped the patients according to the number of previous stent layers at the lesions, an increase in the proportion of PCB angioplasty was observed with the increase in the number of stent layers (*P*_for trend_ = 0.007), as shown in Fig. 3.

### Clinical Outcomes

Over the median follow-up of 1160 (interquartile range 937 to 1536) days and a minimum follow-up time of 383 days, 58 (27.1%) patients had MACEs, mainly driven by TLR (20.6%) (Table 4). There was no significant difference in the incidence of MACEs and their components between the PCB angioplasty group and the DES repeat stenting group during the follow-up (*P* = 0.784). Patients with MACEs were more likely to have CKD (*P* = 0.009), more stent layers in lesions (*P* = 0.001), less time since last stent implantation in target lesion (*P* = 0.048), and a lower proportion of different antiproliferative drugs (*P* = 0.006) (Online Resource 1).

**Table 4:** Clinical outcomes

	PCB (*n* = 75)	DES (*n* = 127)	*P*-value
MACEs, *n* (%)	22 (28.2)	36 (26.5)	0.784
Cardiac death, *n* (%)	2 (2.6)	6 (4.4)	0.713
MI, *n* (%)	11 (14.1)	16 (11.8)	0.620
TLR, *n* (%)	17 (21.8)	27 (19.9)	0.735

Data were presented as *n* (%)

*DES* drug-eluting stent, *PCB* paclitaxel-coated balloon, *MACE* major adverse cardiac event, *MI* myocardial infarction, *TLR* target lesion revascularization

**Fig. 4:**
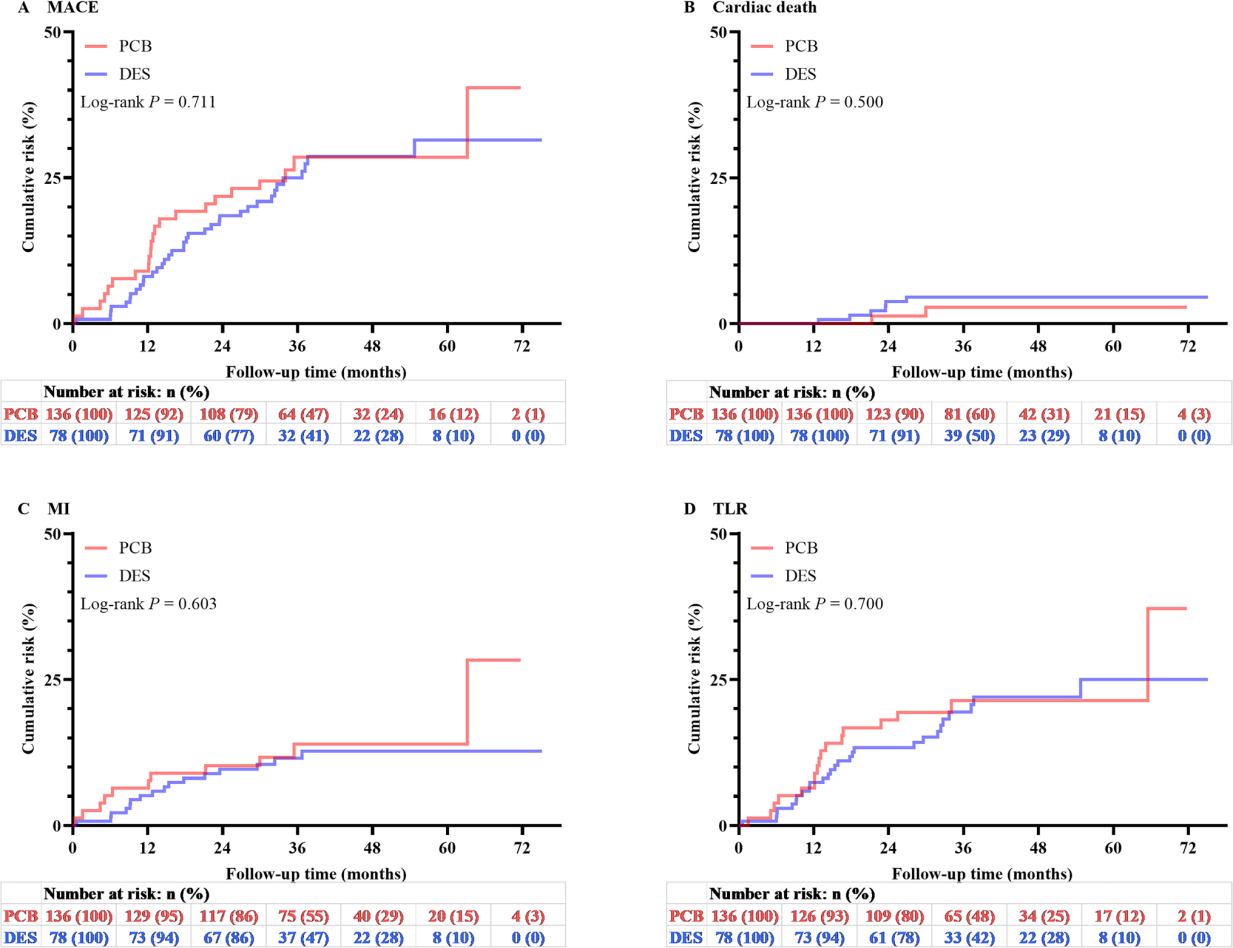
Kaplan–Meier survival curves of incident major adverse cardiac events (**A**), cardiac death (**B**), myocardial infarction (**C**), and target lesion revascularization (**D**) for patients with paclitaxel-coated balloon angioplasty or drug-eluting stent implantation. DES, drug-eluting stent; MACE, major adverse cardiac event; MI, myocardial infarction; PCB, paclitaxel-coated balloon; TLR, target lesion revascularization

Kaplan–Meier curves are shown in Fig. 4. The log-rank test for equality of survivor function showed no significant difference between the PCB angioplasty group and DES repeat stenting group in MACEs (*P* = 0.711) or in cardiac death (*P* = 0.500), MI (*P* = 0.603), and TLR (*P* = 0.700). In the Cox proportional hazards model, different treatments were not associated with the risk of MACEs (HR 1.11, 95% CI 0.65–1.88, *P* = 0.711), even after multifactorial adjustment (HR 1.25, 95% CI 0.64–2.46, *P* = 0.512) (Table 5). Finally, after the adjustment of propensity score–based IPTW (Online Resource 2), there was no significant difference in clinical outcome among different treatment modalities (Online Resource 3). CKD (HR 2.82, 95% CI 1.37–5.83, *P* = 0.005) and ≥ 3 stent layers in lesions (HR 4.50, 95% CI 2.04–9.91, *P* < 0.001) were found to be related to a higher risk of MACEs. Patients treated with different antiproliferative drugs decreased the risk of MACEs by 55% (HR 0.45, 95% CI 0.24–0.85, *P* = 0.014), and the results remained significant for another limus-DES repeat stenting subgroup (HR 0.35, 95% CI 0.15–0.79, *P* = 0.012) (Online Resource 4).

**Table 5:** Univariable and multivariable analyses of MACEs

	Univariable		Multivariable	
	HR (95% CI)	*P*-value	HR (95% CI)	*P*-value
PCB angioplasty	1.11 (0.65–1.88)	0.711	1.25 (0.64–2.46)	0.512
Male	0.89 (0.46–1.72)	0.729	–	–
Age (per 1-year increment)	1.00 (0.97–1.03)	0.803	–	–
Current smoker	0.79 (0.44–1.41)	0.421	–	–
Diabetes	1.54 (0.92–2.57)	0.103	–	–
CKD	2.93 (1.45–5.83)	0.002	2.82 (1.37–5.83)	0.005
Previous MI	1.22 (0.73–2.06)	0.445	–	–
Ejection fraction (per 1% increment)	0.98 (0.96–1.01)	0.146	–	–
Triglycerides (per 1-mmol/l increment)	0.95 (0.75–1.21)	0.685	–	–
High bleeding risk	1.25 (0.63–2.46)	0.528	–	–
DAPT	0.23 (0.06–0.96)	0.044	0.28 (0.07–1.20)	0.086
Left anterior descending artery CTO	Reference		–	–
Left circumflex artery CTO	1.21 (0.71–2.07)	0.481	–	–
Right coronary artery CTO	0.58 (0.20–1.65)	0.306	–	–
Calcification	1.45 (0.87–2.43)	0.158	–	–
CTO lesion length ≥ 20 mm	1.29 (0.71–2.36)	0.403	–	–
Moderate or severe tortuosity	0.63 (0.29–1.39)	0.252	–	–
Ostial lesion	1.02 (0.61–1.71)	0.937	–	–
Proximal cap side branch	1.12 (0.66–1.89)	0.670	–	–
Diseased distal landing zone	1.01 (0.64–1.80)	0.796	–	–
Time since last stents implantation in target lesion (per 1-month increment)	0.99 (0.99–1.00)	0.057	0.99 (0.99–1.00)	0.079
1 stent layer	Reference		Reference	
2 stent layers	1.92 (1.09–3.39)	0.024	1.55 (0.86–2.81)	0.146
≥ 3 stent layers	4.28 (2.04–8.99)	<0.001	4.50 (2.04–9.91)	<0.001
Different antiproliferative drugs	0.51 (0.31–0.86)	0.011	0.45 (0.24–0.85)	0.014

The data are presented as the hazard ratio (95% confidence interval) and *P* value

*CI* confidence interval, *CKD* chronic kidney disease, *CTO* chronic total occlusion, *DAPT* dual antiplatelet therapy, *HR* hazard ratio, *MI* myocardial infarction, *PCB* paclitaxel-coated balloon

## Discussion

The main findings of our study are as follows:

1. With the increase in the number of previous stent layers in DES IS-CTO lesions, the proportion of PCB gradually increased.
2. After successful revascularization with PCB or DES in patients with DES IS-CTO, MACEs occurred in approximately one-fourth of patients during the median follow-up of 3 years and were mainly driven by TLR.
3. No significant difference in long-term clinical outcomes was observed between patients who underwent PCB angioplasty and those who underwent DES repeat stenting in the treatment of DES IS-CTO.
4. CKD and ≥ 3 stent layers in the lesion were independent predictors of adverse prognosis, while switching to another antiproliferative drug was an independent protective factor.

For a long time, ISR has been a problem for operators despite the evolution of stents [25]. In the era of DES, CTO occurring within stents has become a unique type of coronary artery disease. Unlike bare-metal stent (BMS) IS-CTO, the main cause of DES IS-CTO is acute thrombotic occlusion followed by neointimal hyperplasia-related restenosis, hypersensitivity, neointimal erosion, neoatherosclerotic rupture, and edge-related disease [26].

In our study, patients with DES IS-CTO were observed to have a high incidence of long-term adverse events. Previous studies have shown that regardless of the therapeutic modality, the clinical outcomes of DES-ISR were worse than those of BMS-ISR [27]. A study of 11,961 CTO PCI cases at 107 centers worldwide reported that interventions for IS-CTOs and de novo CTOs had similar rates of technical success, procedural success, and in-hospital adverse events, but poorer prognosis [28]. Other contemporary studies also confirmed that compared to de novo CTO, IS-CTO was associated with poorer long-term outcomes despite similar procedural success rates [29–31]. The objective of our study was the intersection of the two adverse lesions, DES-ISR, and IS-CTO. Especially in the era of widespread application of DES, DES IS-CTO has become a troublesome type of lesion that operators have to face. However, the poor prognosis, which was not parallel to the high success rate, was an unacceptable fact for operators. Vasodilation is severely impaired after IS-CTO PCI, which leads to hemodynamic changes and promotes the progression of thrombosis or atherosclerosis [32]. The phenomenon of multilayered stents at lesions was observed in nearly half of the cases in our study. Previous studies have found that multilayer stent implantation may be associated with an increased risk of abnormal vascular response and stent recoiling leading to underexpansion, which in turn leads to a worse long-term prognosis [30, 33].

In the published literature, few studies have specifically explored the long-term outcomes of PCB or DES treatment with IS-CTO. Basavarajaiah et al. [34] compared the long-term outcome of 403 IS-CTO patients treated with balloon angioplasty (BA), drug-coated balloon (DCB), or DES. During the 4-year median follow-up period, MACEs occurred in about half of the patients, of which the BA group had the worst outcome. Compared with the other two treatment modalities, the DCB group had a lower rate of revascularization, but the difference was not statistically significant. In our study, no significant difference in long-term clinical events was observed between PCB angioplasty group and DES repeat stenting group for DES IS-CTO. As the two most effective treatments for ISR, the question about PCB angioplasty and DES repeat stenting which one is better has always been the focus of academic attention. In the 2018 European Society of Cardiology/European Association for Cardiothoracic Surgery Guidelines on Myocardial Revascularization, both DESs and DCBs were recommended for the treatment of ISR, whether BMS-ISR or DES-ISR (Class I, Level of Evidence: A) [10]. A large meta-analysis of 10 randomized clinical trials showed that angioplasty with PCB was less effective than repeat stenting with DES in reducing TLR in the treatment of DES-ISR [11].

As an alternative implant, DCBs are coated with antiproliferative drugs based on a lipophilic matrix, such as paclitaxel, which homogeneously transfers the drug to the coronary artery wall during balloon dilatation. The obvious advantage of DCBs is that they do not require additional metal layers, and they prevent the risks associated with permanent implants and reduce the incidence of inflammation and thrombosis. However, the disadvantages of intervention without implantation are the reduction in acute gain and occurrence of acute recoil [12]. As the other choice of the two main treatment options, DES stenting seems to be the more common and widely accepted treatment for CTO. However, repeated stenting in IS-CTO may again trigger previous stent restenosis factors, such as local abnormal inflammation, adverse reactions to the stent polymer, resistance to antiplatelet agents, stent malapposition or underexpansion, and even immune diseases [29, 35]. Therefore, in the intervention of DES IS-CTO, especially in the treatment of complex lesions with multiple stent layers, there is a dilemma that every operator must face. Although the placement of PCBs can prevent the need for additional stent placement, it does not bring satisfactory long-term outcomes. Similarly, although DES can bring good acute and midterm outcomes, it constitutes a part of the vicious circle of ISR in the long run [36].

A phenomenon of routine intervention for DES IS-CTO has been observed: as the number of stent layers increased in the lesions, operators preferred PCB angioplasty as an emerging alternative option to additional stent implantation. The current theory holds that stent overlap increases the risk of restenosis and recurrent ISR, and additional stenting at ISR lesion can further impair muscle reactivity and endothelial function [37]. In a number of large multicenter registries published in recent years, stenting has been found to be less used in the interventions of IS-CTOs than in de novo CTOs [28]. This may represent the operator’s concern that repeated stenting may be associated with poor prognosis. Our study confirmed the operator’s concern that the long-term outcomes of IS-CTO with multilayer metal stents were worse than those with single-layer metal stents. Yabushita et al. [33] presented data from the New Tokyo Registry that evaluated the clinical outcome of DCBs for the treatment of ISR based on the number of previous metallic layers. MACEs and TLR at 1 year after DCB treatment were significantly higher in patients with ≥ 3 stent layers than in those in the 1 and 2 stent layer groups, and ≥ 3 metallic layers were independent predictors of MACEs. For multimetal layer ISR, previous stent underexpansion or stent fracture may be more difficult to correct, which may be a possible reason for the poor prognosis of multimetal layer ISR. Since multilayer metal stents are associated with poor prognosis, operators should avoid placing stents with more than 2 layers. For patients with symptomatic recurrent restenosis, other alternative treatments may be considered, such as DCB, brachytherapy, or bypass grafting [38].

In this study, CKD was independently associated with poor prognosis, which was expected, as a higher incidence of MACEs was also observed in a subset of patients with CKD complicated with ISR in previous studies [39]. In the International Study of Comparative Health Effectiveness with Medical and Invasive Approaches-Chronic Kidney Disease trial, revascularization did not reduce the risk of death and nonfatal MI in patients with stable coronary heart disease and advanced CKD [40]. Since each intervention causes additional damage to renal function, current guidelines recommend that the contrast load greater than 4 times estimated glomerular filtration rate should be used as an indication for terminating CTO-PCI attempts [41], leaving very limited room for physicians to operate in patients with CKD. Therefore, operators should carefully weigh the risks and benefits and be cautious when considering interventions for such patients. It must be emphasized that the effective treatment cannot be achieved without comprehensive and integrated management of individuals. Therefore, for patients with DES IS-CTO complicated with CKD, in addition to the necessary interventional therapy and antiplatelet therapy, active control of clinical complications and guideline-directed medical therapy may be helpful for improving prognosis [38].

We observed that switching to another antiproliferative drug was associated with better clinical outcomes. In our study, almost all PCB angioplasties adopted a switching strategy, which may be because fewer patients were previously implanted with paclitaxel-eluting stents in our study. Therefore, we analyzed limus-DES as a subgroup and confirmed that the switching to another antiproliferative drug was still significantly associated with a better prognosis in the DES repeat stenting group. In the Restenosis Intra-Stent: Balloon Angioplasty Versus Drug-Eluting Stent trial, a prospective multicenter study evaluated the angiographic and clinical outcomes of DES with different drug or alternative interventional modalities in the treatment of DES-ISR [42]. The main finding was that treatment of DES-ISR with different DESs (switch strategy) was associated with better angiographic and clinical long-term results. When compared in the cohort that underwent repeat stenting, the angiographic results of different DES approaches were superior to those of the same DES approach, which also showed a better trend in clinical results. The theoretical basis of DESs with different antiproliferative drugs in the treatment of DES-ISR is based on the different mechanisms of action of their active pharmacologic agents. Tissues respond differently to different DESs, so the factors involved in previous stent failure may be corrected by switching to DESs with different antiproliferative drugs [43]. Although there are many different mechanisms involved in ISR, when the previous DES fails, operators should be cautious in developing new intervention strategies, in which case switching to another antiproliferative drug may be an attractive treatment strategy.

The classification of ISR has been updated in recent years. In the past, Mehran et al. [44] developed a classification of ISR based on BMS according to the characteristics of angiography. With the promotion of DES and an in-depth understanding of the restenosis mechanism, Waksman et al. [45] based on the mechanism of restenosis proposed a new classification for DES-ISR. Sekiguchi et al. [46] proposed four occlusion patterns of IS-CTO and found differences in the technical success rate, guidewire crossing times, and crossing strategy among different patterns. In the DES era, both the correct classification and individualized treatment of DES IS-CTO based on the mechanism and occlusion patterns of restenosis are crucial to improve clinical efficacy. It is challenging to infer the mechanism of stent failure by angiography alone, and intracoronary imaging can effectively identify the mechanical and biological mechanisms of ISR [22]. Intravascular ultrasound (IVUS)-guided IS-CTO PCI can assist in the selection of appropriate stents, optimize the final result, and provide acceptable long-term clinical outcomes [8]. In addition, optical coherence tomography (OCT) has been recommended by the European Association of Percutaneous Cardiovascular Interventions, as the preferred imaging technique for studying ISR [47]. OCT can provide higher resolution images for the evaluation of morphological features, thus helping to correct factors related to past failures and optimize the intervention [48]. Although previous stents can serve as a roadmap for intervention, tracking through the stented segment theoretically reduces the risk of dissection or injury during the procedure, further improving the procedure success rate [30]. In routine recanalization practice, however, conditions such as stent underexpansion or stent rupture often make wiring more difficult, and the complexity of the IS-CTO means presence of subintimal or extra-stent wire passage, difficulty in device delivery, and even crushing the occluded stent, which increases the risk of adverse events [28, 49, 50]. Recently, the IS-CTO score system was established to predict the technical success of IS-CTO PCI via antegrade approach, which reflects the academic circle’s attention to this special type of coronary artery disease [50]. We are pleased to see that with the improvement of technology, the innovation of CTO PCI devices, and the accumulation of experience, the success rate of contemporary IS-CTO PCI is increasing [28]. Of course, the best way to solve DES IS-CTO is to prevent it. Once the DES IS-CTO happens, trying to identify the cause behind it, correct the undesirable factors, and avoid repeating the same mistakes is the direction we should strive for.

### Limitations

This study has several limitations. First, this was a single-center, retrospective study of a small cohort with inevitable shortcomings. Second, because the choices of implants were determined by the operators or interventional cardiologist teams, there was selection bias in this real-world study. Although we performed propensity score to minimize possible bias, larger multicenter randomized controlled trials are still needed to clarify the clinical outcome of DES IS-CTO. Third, the proportion of intracoronary imaging used in our study was much lower than in other large IS-CTO studies conducted at the same period [28], so it was not possible to carefully evaluate the pathological features and potential mechanism of DES IS-CTO, and the lack of an IVUS- or OCT-optimized intervention may be associated with poor clinical outcomes; therefore, the results of this study should be interpreted carefully. Fourth, this study only recorded the medication of patients at discharge, but the medication of patients after discharge, including dose, frequency, and duration, has not been well evaluated. Therefore, it was not clear whether drug factors affect the long-term prognosis of patients. Last, this study excluded patients with unknown data on previous interventional procedures, so the conclusions of this study may not be extended to all patients with DES IS-CTO.

## Conclusions

PCB angioplasty for the treatment of DES IS-CTO is effective and has similar long-term outcomes to DES repeat stenting in contemporary practice. Successful DES IS-CTO interventions were accompanied with a higher rate of long-term adverse events, especially for patients with CKD and multilayered stents. Switching to another antiproliferative drug may provide better long-term clinical outcomes, especially for patients with limus-DES repeat stenting.

### Supplementary Information

Below is the link to the electronic supplementary material.

Supplementary file1 (DOCX 43 KB)

Supplementary file2. Online Resource 2. The SMD of characteristic among IPTW unweighted and weighted sample. CKD: chronic kidney disease; CTO: chronic total occlusion; IPTW: inverse probability of treatment weighting; SMD: standardized mean differences (PNG 133 kb)

High resolution image (TIF 815 kb)

## Data Availability

The raw data supporting the conclusion of this article will be provided by the communication author under reasonable requirements.
