# Supplementary material for: Clinical Outcome of Paclitaxel-Coated Balloon Angioplasty Versus Drug-Eluting Stent Implantation for the Treatment of Coronary Drug-Eluting Stent In-Stent Chronic Total Occlusion
Source: Cardiovasc Drugs Ther. 2022 Aug 5;37(6):1155–66. doi: 10.1007/s10557-022-07363-7 (PMC10721670; doi:10.1007/s10557-022-07363-7)
Supplement: Supplementary file 1 — Supplementary file1 (DOCX 43 KB) [file 10557_2022_7363_MOESM1_ESM.docx]

**Online Resource 1. Demographic, clinical, angiographic and procedural characteristics of patients who did and did not present with MACEs during follow-up**

|  | MACEs  (n=58) | Non-MACEs  (n=156) | *P*-value |  |
| --- | --- | --- | --- | --- |
| Demographic and clinical characteristics | | | | |
| Male, n (%) | 47 (81.0) | 132 (84.6) | 0.529 |  |
| Age, years | 59.4 ± 8.5 | 59.0 ± 9.0 | 0.813 |  |
| Body mass index, kg/m^2^ | 26.5 ± 2.9 | 26.3 ± 3.0 | 0.689 |  |
| Current smoker, n (%) | 16 (27.6) | 52 (33.3) | 0.422 |  |
| Hypertension, n (%) | 35 (60.3) | 99 (63.5) | 0.675 |  |
| Diabetes, n (%) | 30 (51.7) | 64 (41.0) | 0.161 |  |
| Dyslipidemia, n (%) | 44 (75.9) | 119 (76.3) | 0.949 |  |
| CKD, n (%) | 10 (17.2) | 9 (5.8) | 0.009 |  |
| Previous MI, n (%) | 33 (56.9) | 80 (51.3) | 0.465 |  |
| Previous CABG, n (%) | 3 (5.2) | 7 (4.5) | ﹥0.999 |  |
| Ejection fraction, % | 59 (53, 65) | 60 (54, 66) | 0.151 |  |
| Total cholesterol, mmol/l | 3.65 (3.16, 4.31) | 3.75 (3.24, 4.26) | 0.529 |  |
| LDL cholesterol, mmol/l | 2.04 (1.61, 2.64) | 2.06 (1.72, 2.66) | 0.767 |  |
| HDL cholesterol, mmol/l | 0.99 (0.82, 1.16) | 1.02 (0.90, 1.18) | 0.316 |  |
| Triglycerides, mmol/l | 1.37 (1.02, 2.04) | 1.38 (1.06, 2.03) | 0.749 |  |
| Creatinine clearance, ml/min | 99.23 ± 28.99 | 101.15 ± 25.68 | 0.640 |  |
| High bleeding risk, n (%) | 10 (17.2) | 21 (13.5) | 0.515 |  |
| Medications, n (%) |  |  |  |  |
| DAPT | 56 (96.6) | 156 (100.0) | 0.073 |  |
| Statin | 58 (100.0) | 155 (99.4) | ﹥0.999 |  |
| β-Blocker | 42 (72.4) | 115 (73.7) | 0.848 |  |
| ACE-inhibitor/ARB | 30 (51.7) | 68 (43.6) | 0.288 |  |
| Angiographic characteristics | | | | |
| Multivessel disease, n (%) | 29 (50.0) | 97 (62.2) | 0.108 |  |
| CTO target vessel, n (%) |  |  | 0.287 |  |
| Left anterior descending artery | 27 (46.6) | 60 (38.5) |  |  |
| Left circumflex artery | 4 (6.9) | 22 (14.1) |  |  |
| Right coronary artery | 27 (46.6) | 74 (47.4) |  |  |
| J-CTO score | 2 (1, 3) | 2 (1, 3) | 0.577 |  |
| Blunt stump, n (%) | 30 (51.7) | 92 (59.0) | 0.341 |  |
| Calcification, n (%) | 27 (46.6) | 55 (35.3) | 0.131 |  |
| Bending >45°, n (%) | 19 (32.8) | 50 (32.1) | 0.922 |  |
| CTO lesion length ≥20 mm, n (%) | 44 (75.9) | 110 (70.5) | 0.439 |  |
| Reattempt, n (%) | 12 (20.7) | 29 (19.6) | 0.729 |  |
| PROGRESS CTO score | 1 (0, 1) | 1 (0, 2) | 0.178 |  |
| Proximal cap ambiguity, n (%) | 10 (17.2) | 44 (28.2) | 0.101 |  |
| Absence of interventional collaterals, n (%) | 24 (41.4) | 54 (34.6) | 0.361 |  |
| Moderate or severe tortuosity, n (%) | 7 (12.1) | 30 (19.2) | 0.218 |  |
| Circumflex CTO, n (%) | 4 (6.9) | 22 (14.1) | 0.152 |  |
| Ostial lesion, n (%) | 27 (46.6) | 73 (46.8) | 0.975 |  |
| Proximal cap side-branch, n (%) | 24 (41.4) | 60 (38.5) | 0.698 |  |
| Diseased distal landing zone, n (%) | 27 (46.6) | 68 (43.6) | 0.698 |  |
| Collateral 2-3 grade, n (%) | 39 (67.2) | 110 (70.5) | 0.644 |  |
| Time since last stents implantation in target lesion, months | 60 (27, 108) | 74 (46, 110) | 0.048 |  |
| Prior type of DES, n (%) |  |  | 0.681 |  |
| Sirolimus-eluting stent | 40 (69.0) | 110 (70.5) |  |  |
| Everolimus-eluting stent | 5 (8.6) | 16 (10.3) |  |  |
| Zotarolimus-eluting stent | 9 (15.5) | 25 (16.0) |  |  |
| Paclitaxel-eluting stent | 4 (6.9) | 5 (3.2) |  |  |
| Prior stent length, mm | 33 (23, 45) | 32 (23, 48) | 0.909 |  |
| Prior minimum stent diameter, mm | 2.75 (2.50, 3.00) | 2.75 (2.50, 3.00) | 0.836 |  |
| Prior maximum stent diameter, mm | 3.00 (2.75, 3.00) | 3.00 (2.75, 3.00) | 0.607 |  |
| Prior mean stent diameter, mm | 2.88 (2.75, 3.00) | 2.88 (2.63, 3.00) | 0.727 |  |
| Number of stent layers in lesions, n (%) |  |  | 0.001 |  |
| 1 layer | 24 (41.4) | 101 (64.7) |  |  |
| 2 layers | 24 (41.4) | 48 (30.8) |  |  |
| ≥3 layers | 10 (17.2) | 7 (4.5) |  |  |
| Procedural characteristics | | | | |
| Radial access, n (%) | 50 (86.2) | 134 (85.9) | 0.954 |  |
| Dual catheter injection, n (%) | 21 (36.2) | 54 (34.6) | 0.828 |  |
| Primary strategy, n (%) |  |  | ﹥0.999 |  |
| AWE | 55 (94.8) | 149 (95.5) |  |  |
| RWE | 3 (5.2) | 7 (4.5) |  |  |
| Final strategy, n (%) |  |  | 0.531 |  |
| AWE | 55 (94.8) | 141 (90.4) |  |  |
| ADR | 1 (1.7) | 3 (1.9) |  |  |
| RWE | 2 (3.4) | 12 (7.7) |  |  |
| Use of CrossBoss, n (%) | 2 (3.4) | 8 (5.1) | 0.732 |  |
| Use of IVUS, n (%) | 5 (8.6) | 20 (12.8) | 0.395 |  |
| Use of OCT, n (%) | 2 (3.4) | 6 (3.8) | ﹥0.999 |  |
| Total procedure time, min | 80 (61, 105) | 88 (65, 108) | 0.196 |  |
| Total fluoroscopy time, min | 28 (20, 37) | 30 (22, 42) | 0.231 |  |
| Total contrast volume, ml | 160 (140, 200) | 180 (140, 210) | 0.164 |  |
| Use of PCB, n (%) | 22 (37.9) | 56 (35.9) | 0.784 |  |
| Number of PCB, n | 1 (1, 3) | 1 (1, 2) | 0.086 |  |
| Total PCB length, mm | 48 (30, 71) | 30 (27, 52) | 0.122 |  |
| Minimum PCB diameter, mm | 2.50 (2.50, 3.00) | 3.00 (2.50, 3.00) | 0.476 |  |
| Maximum PCB diameter, mm | 3.00 (2.50, 3.50) | 3.00 (2.50, 3.38) | 0.800 |  |
| Mean PCB diameter, mm | 2.75 (2.50, 3.06) | 3.00 (2.50, 3.00) | 0.548 |  |
| Maximal PCB pressure, atm | 12 (9, 12) | 10 (8, 12) | 0.290 |  |
| Duration of Inflation, seconds | 60 (50, 60) | 60 (45, 60) | 0.597 |  |
| Use of DES, n (%) | 36 (62.1) | 100 (64.1) | 0.784 |  |
| Number of DES, n | 2 (1, 3) | 2 (1, 2) | 0.241 |  |
| Type of DES, n (%) |  |  | 0.778 |  |
| Sirolimus-eluting stent | 24 (66.7) | 60 (60.0) |  |  |
| Everolimus-eluting stent | 8 (22.2) | 27 (27.0) |  |  |
| Zotarolimus-eluting stent | 4 (11.1) | 13 (13.3) |  |  |
| Total stent length, mm | 60 (33, 88) | 54 (33, 71) | 0.377 |  |
| Minimum stent diameter, mm | 2.75 (2.50, 3.00) | 2.50 (2.50, 3.00) | 0.755 |  |
| Maximum stent diameter, mm | 3.00 (2.75, 3.50) | 3.00 (2.75, 3.50) | 0.622 |  |
| Mean stent diameter, mm | 2.94 (2.63, 3.13) | 2.75 (2.63, 3.13) | 0.548 |  |
| Different antiproliferative drug, n (%) | 30 (51.7) | 112 (71.8) | 0.006 |  |

Data are presented as the mean ± standard deviation, n (%) or median (Q1, Q3). ACE: angiotensin-converting enzyme; ADR: antegrade dissection re-entry; ARB: angiotensin receptor blocker; AWE: antegrade wire escalation; CABG: coronary artery bypass graft; CKD: chronic kidney disease; CTO: chronic total occlusion; DAPT: dual antiplatelet therapy; DES: drug-eluting stent; HDL: high-density lipoprotein; IVUS: intravascular ultrasound; LDL: low-density lipoprotein; MACE: major adverse cardiac event; MI: myocardial infarction; OCT: optical coherence tomography; PCB: paclitaxel-coated balloon; RWE: retrograde wire escalation

**Online Resource 3. Risk of clinical outcome after IPTW**

|  | HR (95% CI) | *P*-value |
| --- | --- | --- |
| MACEs | 0.76 (0.42-1.36) | 0.354 |
| Cardiac death | 0.57 (0.12-2.76) | 0.481 |
| MI | 0.86 (0.37-1.98) | 0.719 |
| TLR | 0.70 (0.36-1.35) | 0.383 |

Reference category is DES repeat stenting. The data are presented as the hazard ratio (95% confidence interval) and *P* value. CI: confidence interval; HR: hazard ratio; IPTW: inverse probability of treatment weighting; MACE: major adverse cardiac event; MI: myocardial infarction; TLR: target lesion revascularization

**Online Resource 4. Univariable and multivariable analyses of MACEs for patients with limus-DES repeat stenting**

|  | MACEs, n (%) | Univariable | | Multivariable | | |
| --- | --- | --- | --- | --- | --- | --- |
|  |  | HR (95% CI) | *P*-value | HR (95% CI) | *P*-value |  |
| Same antiproliferative drug | 26 (38.2) | Reference | | Reference | | |
| Different antiproliferative drug | 8 (12.7) | 0.29 (0.13-0.64) | 0.002 | 0.35 (0.15-0.79) | 0.012 |  |

The data are presented as the hazard ratio (95% confidence interval) and *P* value. Multivariable model was adjusted for final implantation, CKD, dual antiplatelet therapy, time since last stents implantation in target lesion, number of stent layers in lesions, and different antiproliferative drug. CI: confidence interval; DES: drug-eluting stent; HR: hazard ratio; MACE: major adverse cardiac event
