# Supplementary figures and images for: Clinical Outcome of Paclitaxel-Coated Balloon Angioplasty Versus Drug-Eluting Stent Implantation for the Treatment of Coronary Drug-Eluting Stent In-Stent Chronic Total Occlusion
Source: Cardiovasc Drugs Ther. 2022 Aug 5;37(6):1155–66. doi: 10.1007/s10557-022-07363-7 (PMC10721670; doi:10.1007/s10557-022-07363-7)

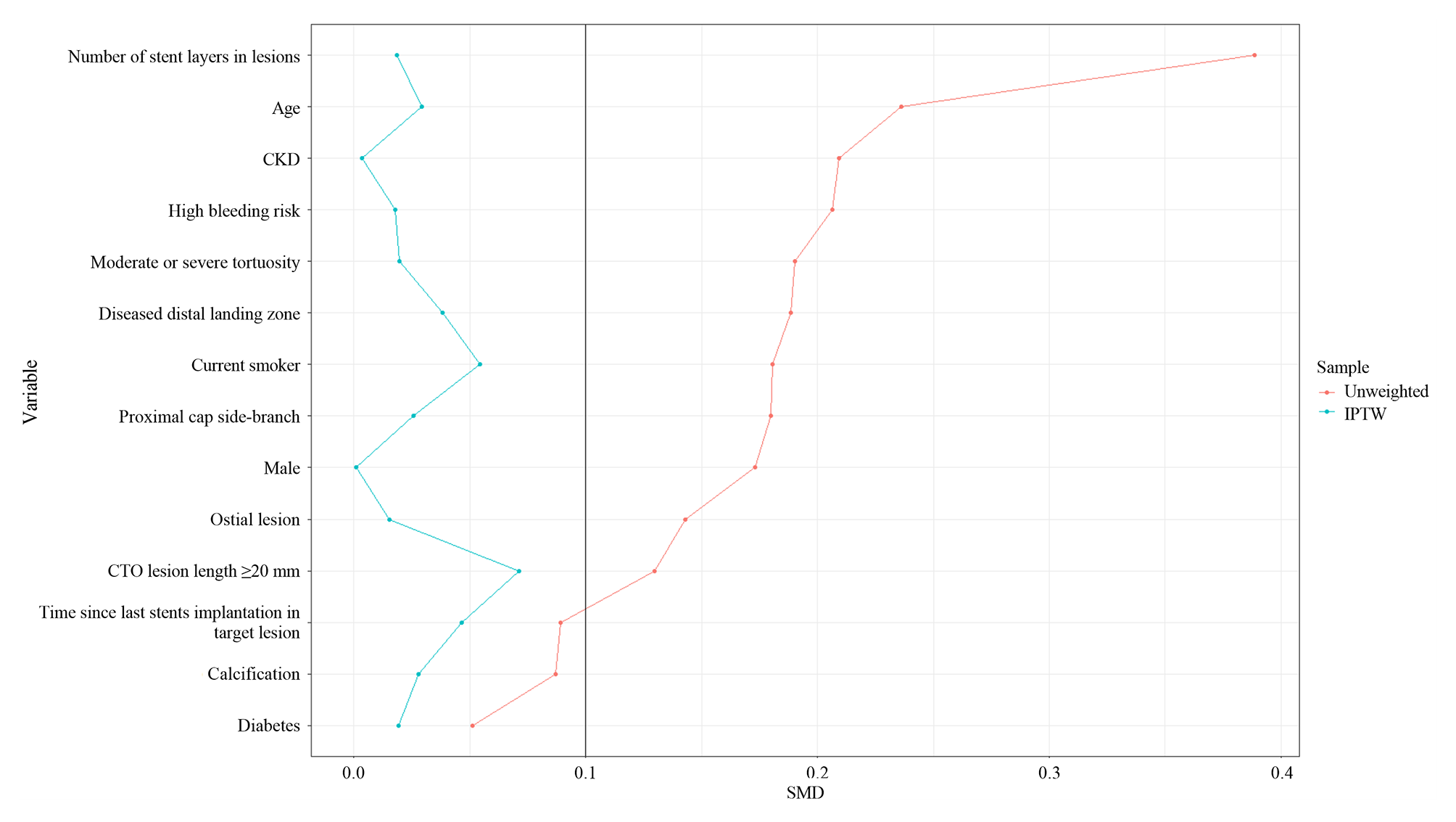

Supplement: Supplementary file 2 — Supplementary file2. Online Resource 2. The SMD of characteristic among IPTW unweighted and weighted sample. CKD: chronic kidney disease; CTO: chronic total occlusion; IPTW: inverse probability of treatment weighting; SMD: standardized mean differences (PNG 133 kb) [file 10557_2022_7363_Fig5_ESM.png]

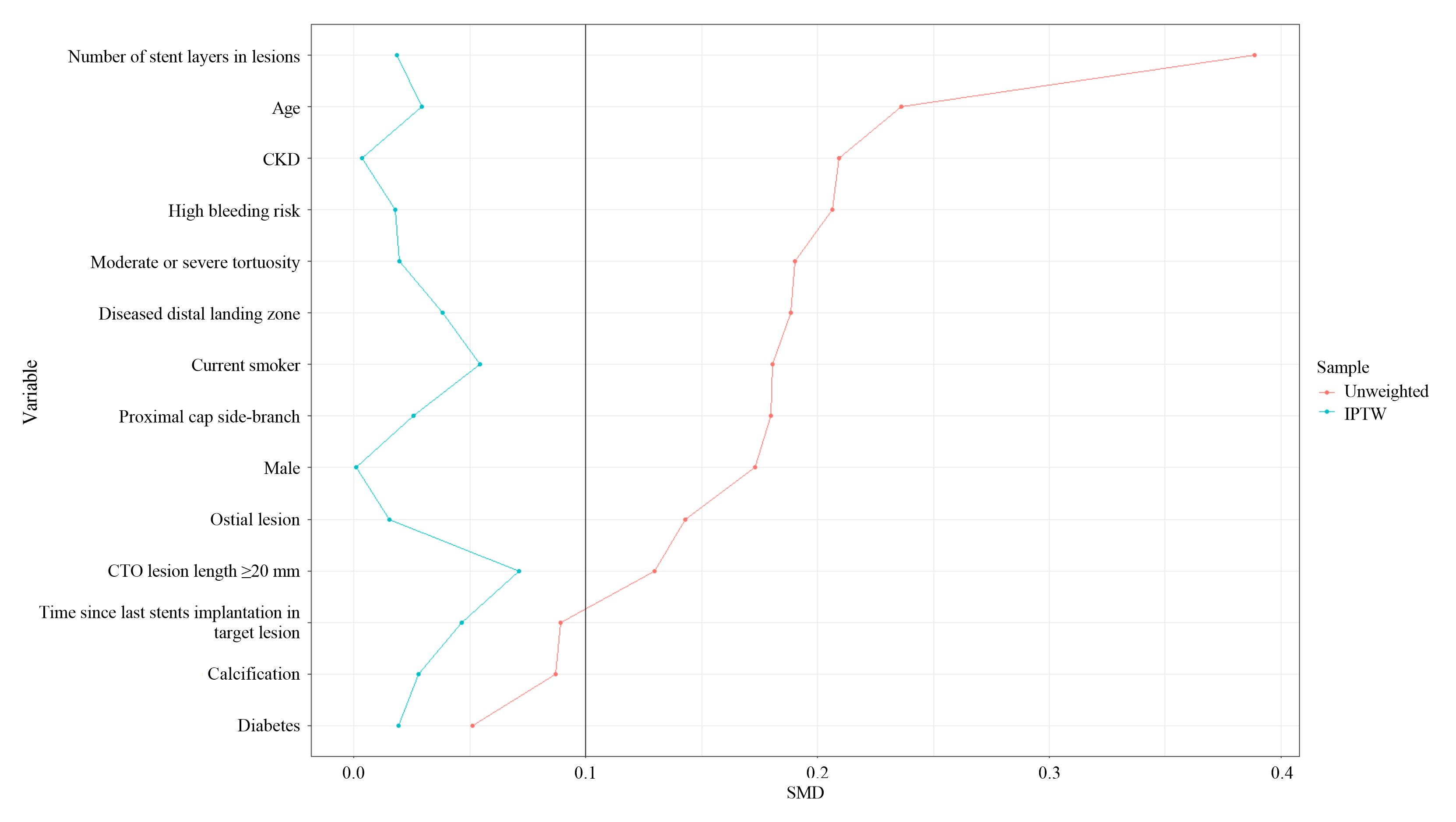

Supplement: Supplementary file 3 — High resolution image (TIF 815 kb) [file 10557_2022_7363_MOESM2_ESM.tif]
